# Clinical and prognostic ^18^F-FDG PET/CT role in recurrent vulvar cancer: a multicentric experience

**DOI:** 10.1007/s11604-021-01173-x

**Published:** 2021-07-17

**Authors:** Domenico Albano, Mattia Bonacina, Giordano Savelli, Paola Ferro, Elena Busnardo, Luigi Gianolli, Luca Camoni, Raffaele Giubbini, Francesco Bertagna

**Affiliations:** 1 Nuclear Medicine, University of Brescia and ASST Spedali Civili Brescia, P.le Spedali Civili, 1, 25123 Brescia, Italy; 2 Nuclear Medicine Department, Fondazione Poliambulanza, Brescia, Italy; 3 Nuclear Medicine Department, IRCCS San Raffaele Scientific Institute, Via Olgettina 60, 20132 Milan, Italy

**Keywords:** Vulvar cancer, ^18^F-FDG PET, CT, PET, CT, Restaging

## Abstract

**Purpose:**

The aim of this retrospective multicentric study was to investigate the diagnostic performance, the prognostic value and the impact of ^18^F-FDG PET/CT on treatment decision-making in patients with suspected recurrent vulvar cancer (VC).

**Materials and methods:**

Sixty-three patients affected by VC performed ^18^F-FDG-PET/CT for restaging purposes in case of suspected clinical and/or radiological recurrence. Histopatology results if available and/or clinical-imaging follow-up for at least 12 months were considered as reference standard. The diagnostic accuracy and the clinical impact of ^18^F-FDG PET/CT were investigated. Progression free survival (PFS) and overall survival (OS) were calculated using Kaplan–Meier curves.

**Results:**

Fifty-two (82.5%) PET/CT showed the presence of recurrence, while the remaining 11 (17.5%) were negative. Sensitivity, specificity, positive predictive value, negative predictive value and accuracy of PET/CT were 100% (95%CI 93–100%), 92% (95%CI 62–100%), 98% (95%CI 89–99%), 100% and 98% (95%CI 92–100%). A relevant impact of ^18^F-FDG PET/CT imaging was registered in 28 cases: in 12 cases moving from local therapy to chemotherapy due to the recognition of disseminate localizations; in 10 showing the site of recurrence in presence of negative conventional imaging, and in 6 cases confirming to be true negative and avoiding unnecessary therapies. Beside advanced age and HPV status, a positive restaging ^18^F-FDG PET/CT scan was significantly correlated with shorter PFS and OS compared to negative scan (*p* < 0.001).

**Conclusions:**

^18^F-FDG PET/CT demonstrated to be an accurate tool in the assessing of recurrent VC with high sensitivity and specificity and with a significant impact on clinical decision-making. Restaging ^18^F-FDG PET/CT findings were associated with survival.

## Introduction

Vulvar cancer (VC) is a rare gynecologic malignancy, with an incidence rate of 1.5–2.4 per 100,000 women per year [1, 2]. Vulvar squamous cell carcinoma is the most common histotype followed by melanoma, basal cell carcinoma, adenocarcinoma and Paget disease [3]. Vulvar squamous cell carcinoma has usually two tumor pathways: a non-Human Papillomavirus (HPV) associate path, common in older women and more aggressive, and a HPV-associated, typical of younger women and with better prognosis [4].

The pattern of dissemination of VC is predominantly lymphogenic to the inguinofemoral lymph nodes, while distant metastases are very rare [5]. The lymph node involvement affects prognosis together with the depth of stromal invasion and the presence of lymph-vascular invasion [5, 6]. The 5-year overall survival (OS) rate ranges from 95% in patients without nodal disease to 61% in women with nodal metastases [6]. Nowadays, the primary treatment for VC includes radical resection of the primary lesion associated with unilateral or bilateral surgical of the inguinofemoral lymph nodes. Adjuvant radiotherapy is usually performed in case of other coexistent risk factors or aggressive disease [7]. 18-fluorodeoxyglucose positron emission tomography/computed tomography (^18^F-FDG PET/CT) is a non-invasive imaging tool which showed high accuracy in the preoperative workup and staging of VC, especially presenting a high negative predictive value [8]. Instead, in the restaging field, ^18^F-FDG PET/CT role is yet unclear with only promising findings [9, 10]; but these studies are affected by several limitations, such as the heterogeneous population studied (vaginal and vulvar cancers often analyzed together) and the limited sample of patients included. The current restaging and follow-up strategy of VC consists of conventional imaging (CI) studies, including ultrasonography, computed tomography (CT) and/or magnetic resonance imaging (MRI) [11, 12], but these tools may have a limited detection rate in identifying recurrence. One of the major issue is to discriminate between pathological tissue and fibrotic-necrotic tissue after treatment. In this field, ^18^F-FDG PET/CT has proven to be useful in different gynecological malignancies, mainly if PET/CT scan is performed after an appropriate interval time after treatments [13, 14]. The metabolic-functional change may anticipate the morphological-radiological modification and this is the reason why PET/CT may theoretically evaluate earlier treatment response and recognize recurrence better than CI. An early detection of VC recurrence may help to stratify better the patients and start therapies early with a potential positive impact on outcome survival.

The aim of this retrospective multicentric study was to investigate the diagnostic performance, the prognostic value and the impact of ^18^F-FDG PET/CT on treatment decision-making in a sample of patients affected by VC and suspected recurrence.

## Materials and methods

This study was approved by ethics committee (NP 3693) of ASST Spedali Civili Hospital of Brescia. We have retrospectively screened all patients who performed a ^18^F-FDG PET/CT scan in three Nuclear Medicine Units from December 2009 to December 2019. Among this large group of patients, 63 women (average age: 66 years; age range, 31–89) had a history of VC with histological confirmation and underwent a ^18^F-FDG PET/CT scan for restaging purpose in presence of suspected recurrence (Table 1). We reviewed and analyzed the main epidemiological (age at diagnosis, The International Federation of Gynecology and Obstetrics (FIGO) stage, HIV status, HPV status), morphological (tumor size) and histological (histotype) data, metabolic features derived by ^18^F-FDG PET/CT and follow-up data. All patients except four were treated with local surgery for primary tumor: a simple partial vulvectomy was performed in 23 patients, a complete vulvectomy in 36. In 25 cases, vulvectomy was associated with unilateral groin dissection, while in 14 with bilateral groin dissection. Post-surgical adjuvant radiotherapy was done in 20 patients; only radiotherapy as primary treatment in 4 women. A combination of surgery, chemotherapy and radiotherapy was performed in 5 cases. At the time of diagnosis, 18 patients were in stage I according to FIGO classification, 7 in stage II, 29 in stage III and 9 in stage IV. The most frequent VC histotype was squamous cell carcinoma in 58 patients, follow by adenocarcinoma in 3 cases and melanoma in 2. Recurrence was suspected based on the clinical suspicion in 17 women and because of imaging findings on CI (MRI, CT) exams in 46 women.

**Table 1:** Baseline characteristics of 63 patients

	Frequency	%
Age average (range)	66.8 (31–89)	
Primary FIGO stage		
I	18	29
II	7	11
III	29	46
IV	9	14
Histotype		
Squamous cell carcinoma	58	92
Adenocarcinoma	3	5
Melanoma	2	3
HIV status		
Positive	5	
Negative	58	
HPV status		
Positive	15	
Negative	48	
Tumor size, mm average (range)	25.1 (4–70)	
Nodal disease at diagnosis	35	56
Treatment		
Surgery	34	54
Surgery + radiotherapy	20	32
Unilateral groin dissection	25	40
Bilateral groin dissection	14	22
Radiotherapy	4	6
Surgery + radiotherapy + chemotherapy	5	8
18F-FDG PET/CT result		
Positive	52	82.5
Negative	11	17.5

Average interval time from the end of primary therapy and restaging PET/CT scan was 9 months (range: 6–36) and all PET/CT scans were performed at least 120 days after the end of treatments.

### ^18^F-FDG PET/CT features

^18^F-FDG PET/CT was performed after at least 6 h fasting and with the glucose level lower than 150 mg/dl. An activity of 3.5–4.5 MBq/Kg of ^18^F-FDG was injected intravenously and PET/CT scan was acquired 60 ± 10 min after the radiotracer injection from the skull basis to the mid-thigh (3–4 min per bed-PET-step of 15 cm). The PET/CT devices used were: a Discovery STE PET/CT tomograph (General Electric Company-GE-Milwaukee, WI, USA), a Discovery 690 tomograph (General Electric Company-GE-Milwaukee, WI, USA) and a Biograph mCT tomograph (Siemens Healthineers, Malvern, PA, USA) with standard CT parameters. The reconstruction was performed in a 256 × 256 matrix and field of view of 60 cm. The D-STE acquisition parameters were: 120 kV, fixed tube current 80 mA, 8 slices × 3.75 mm and 3.27 mm interval, pitch 1:5, tube rotation 0.8 s. The D-690 acquisition parameters were: 120 kV, fixed tube current 80 mA, 64 slices × 3.75 mm and 3.27 mm interval, pitch 0.984:1, tube rotation 0.5 s. The Biograph mCT acquisition parameters were: 120 kV, fixed tube current 80 mA, 64 slices × 3.75 mm and 3.27 mm interval, pitch 0.984:1, tube rotation 0.5 s.

For D-690 and Biograph mCT time-of-flight (TOF) and point spread function (PSF) were used as reconstruction algorithms; filter cutoff 5 mm, 18 subsets; 3 iterations. For D-STE ordered subset expectation maximization (OSEM) was applied; filter cutoff 5 mm; 21 subsets, 2 iterations.

The PET images were analyzed visually and semi-quantitatively by measuring the mean and maximum standardized uptake value body weight (SUVmean and SUVmax) of the lesion with the highest uptake. Local nuclear medicine physicians (DA, MB, PF) with experience in 18F-FDG PET /CT evaluation reviewed the images. The readers had knowledge of clinical history, and every focal radiotracer uptake different from physiological distribution and background was judged as suggestive of disease. The readers measured the SUV of detectable lesions by drawing a region of interest (ROI) over the area of maximum activity and the lesion with the highest 18F-FDG uptake in each patient was taken as reference lesion. Moreover, SUVmax of the liver was calculated at the VIII hepatic segment of axial PET images using a round-shape 10 mm ROI and SUVmax of the blood-pool was calculated at the aortic arch by use of axial PET images with a round-shape 10 mm ROI not involving the vessel wall. Then, the ratios between SUVmax of the hypermetabolic lesion and SUVmax of the liver and blood-pool of each patient was calculate to obtain lesion to liver SUVmax ratio (L-L SUV R) and lesion to blood-pool SUVmax ratio (L-BP SUV R).

The subsequent histopathological confirmation or clinical-imaging information during follow-up after ^18^F-FDG PET/CT were considered the standard of reference. Verification by surgical exploration or imaging-guided biopsy was performed whenever possible. Otherwise, the ^18^F-FDG PET/CT results were confirmed by radiological or clinical outcomes, especially in patients with disseminate disease. Patients with positive ^18^F-FDG PET/CT results were considered as true-positives (TP) if further evaluations confirmed the malignancy of the lesions and false-positive (FP) if further analysis showed no malignancy. Patients with negative ^18^F-FDG PET/CT scans were judged as true negative (TN) if further examinations revealed no malignancy and false-negative (FN) if further examinations detected malignant ones.

### Evaluation of clinical impact of 18F-FDG PET/CT

The clinical impact of ^18^F-FDG PET/CT was categorized as positive if the additional lesions detected only by PET/CT (and not revealed by other conventional imaging methods, like ultrasonography, MRI and/or CT with or without contrast agent according to the clinical indication) have changed the patient management, or ruled out false-positive findings at conventional imaging avoiding unnecessary therapy or the endorsement of a curative intent. Instead, the clinical impact of PET/CT was evaluated as negative if PET/CT led to unnecessary invasive diagnostic or therapeutic approaches. Moreover, ^18^F-FDG PET/CT findings were labeled as no clinical impact if both ^18^F-FDG PET/CT and conventional imaging results were concordant, or a discordant report between PET/CT and conventional imaging did not affect the subsequent management.

### Statistical analysis

All statistical analysis was carried out using MedCalc 19.3 (Belgium). The descriptive analysis of categorical variables consisted of the calculation of simple and relative frequencies; the numeric variables consisted of mean ± standard deviation (SD), minimum and maximum. A *p* value < 0.05 was considered statistically significant. Using the final diagnosis as a reference, sensitivity (SE), specificity (SP), negative predictive value (NPV), positive predictive value (PPV) and accuracy (AC) were calculated based on Bayes’s law, with 95% confidence intervals (CIs). PFS was determined considering the time interval between restaging PET/CT and the appearance of clinical and/or radiological progression or the last follow-up date. OS was defined as the time interval between restaging PET/CT and death or the last follow-up date. PFS and OS were computed using Kaplan–Meier curves. Univariate and multivariate Cox proportional hazards models were fitted in the whole sample. The relationship between outcome and the variables included was summarized by hazard ratios (HR) with 95% confidence intervals (95% CI).

## Results

### ^18^F-FDG PET/CT performance in restaging

**Fig. 1:**
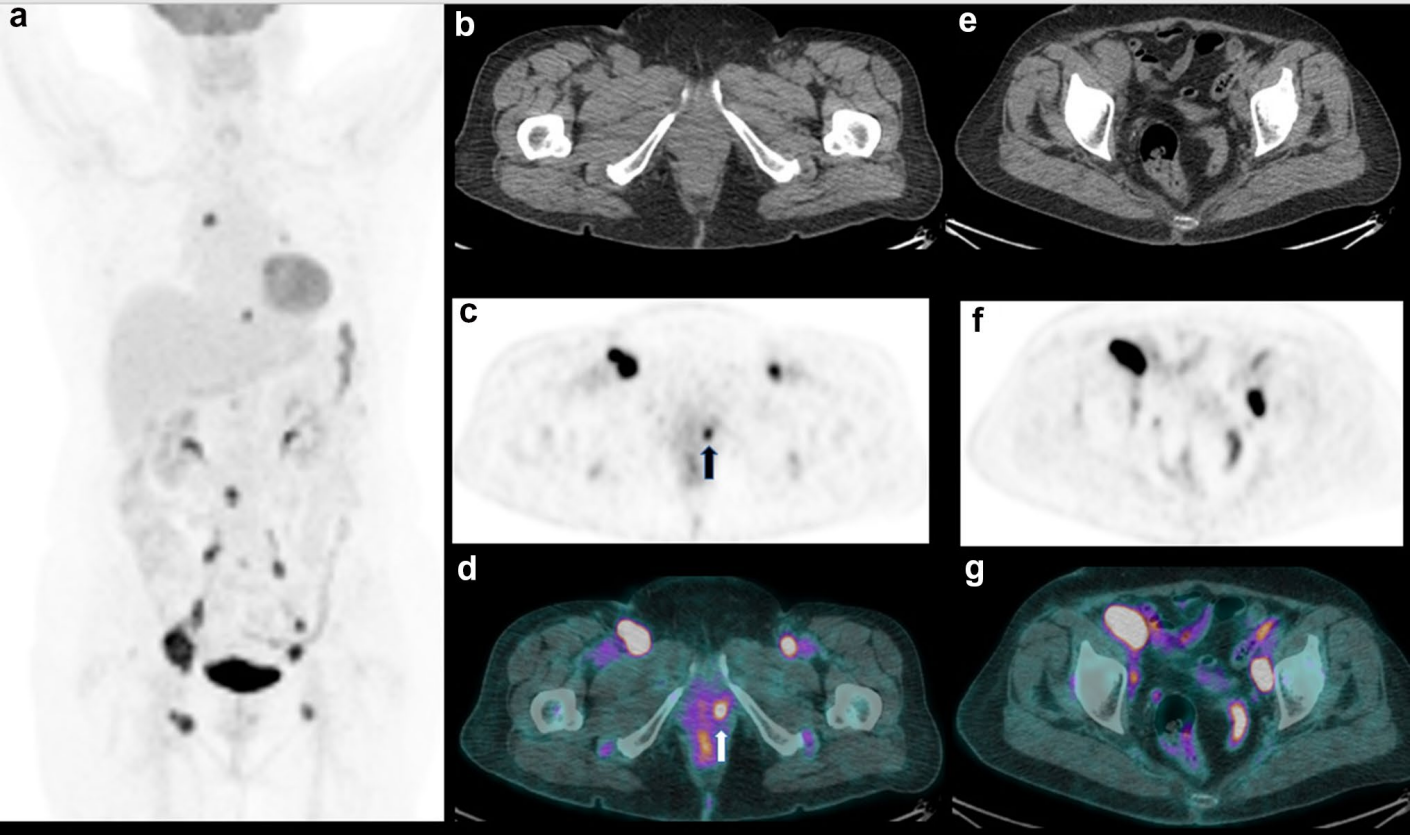
A representative case of positive ^18^F-FDG PET/CT in a 69-year-old woman previously treated with partial vulvectomy. Maximum intensity projection (MIP) (**a**) showing plural increased FDG uptakes in the pelvis, abdomen and chest. Axial CT (**b**), PET (**c)** and PET/CT fused (**d**) images demonstrating uptake corresponding to left vulvar lesion (black arrow) and some bilateral groin nodes. Axial CT (**e**), PET (**f)** and PET/CT fused (**g**) images revealing bilateral iliac nodes

**Fig. 2:**
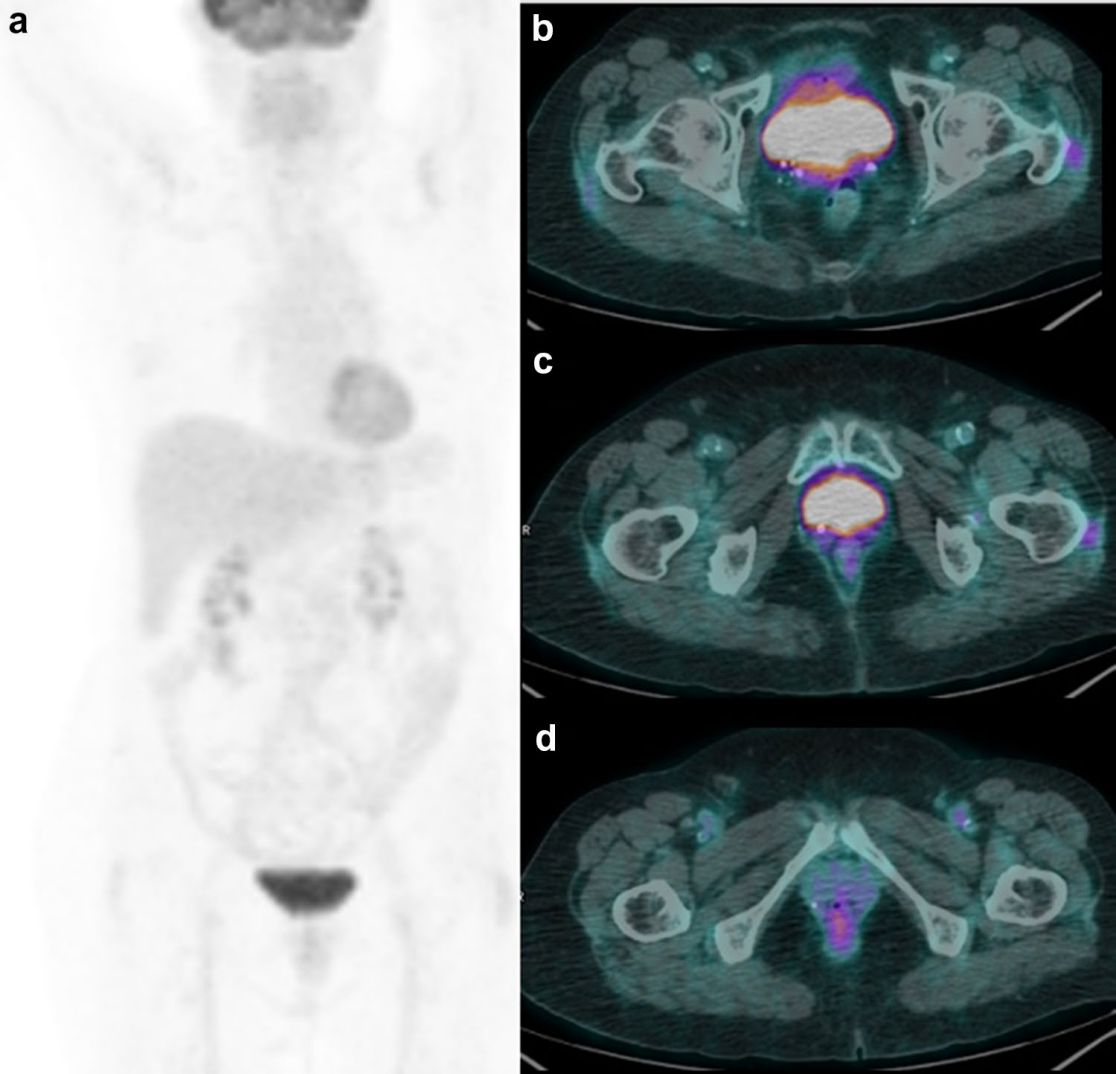
A representative case of true negative ^18^F-FDG PET/CT in a 59-year-old woman with vulvar adenocarcinoma who underwent restaging ^18^F-FDG PET/CT for suspected relapse. The recurrence was suspected by CT, but it was judged negative by PET, and confirmed to be true negative by subsequent follow-up. MIP (**a**) showing no increased FDG uptake in the whole body. Axial PET/CT fused images (**b, c, d**) detecting no pathological uptakes in the pelvis

Comparing with the reference standard described in Methods, a final diagnosis of recurrence/relapse was discovered in 51 (80%) patients at the time of restaging PET/CT, whereas in the other 12 cases no recurrence was considered as definitive diagnosis. The recurrence was confirmed by histological report in 20 patients, whereas in the other 31 the diagnosis was confirmed by clinical and/or imaging follow-up. ^18^F-FDG PET/CT scan showed the presence of FDG-avid disease in 52 (82.5%) patients displaying the presence of at least 1 lesion with increased FDG uptake suspected for local or distant disease, while it was negative in the other 11 scans (17.5%) (Figs. 1, 2).

The localizations of recurrence were local pelvic relapse in 7 cases, locoregional lymph nodes in 19 patients (seven with groin nodes, five with iliac nodes and seven with both inguinal and iliac nodes), both local recurrence and regional lymph nodes in seven patients. Distant metastatic disease in 4 studies, 10 studies showed both locoregional nodes and metastatic disease and 16 local pelvic relapse and distant metastases together. Lung was the most common site of distant metastasis (*n* = 19), followed by peritoneum (*n* = 4), bone (*n* = 3), liver (*n* = 1), liver and bone together (*n* = 4 each).

The average SUVmax and SUVmean of recurrent lesions were 14.5 ± 5.6 (range 5.2–28.6) and 8.7 ± 4.7 (range 2.6–26.1). L-L SUV R and L-BP SUV R were 4.8 ± 2.3 (range 1.1–11) and 6.3 ± 3.3 (range 1.7–15).

According to the standard or reference, on a study-based analysis 51 ^18^F-FDG-PET/CT scans were TP, 11 were TN, 1 was FP and no cases of FN. Therefore, SE, SP, PPV, NPV and AC of ^18^F-FDG-PET/CT in detecting recurrent VC resulted in 100% (CI 93–100%), 92% (CI 62–100%), 98% (CI 89–100%), 100% and 98% (CI 91–100%), respectively. Positive and negative likelihood ratios were 12.00 and 0.00.

**Fig. 3:**
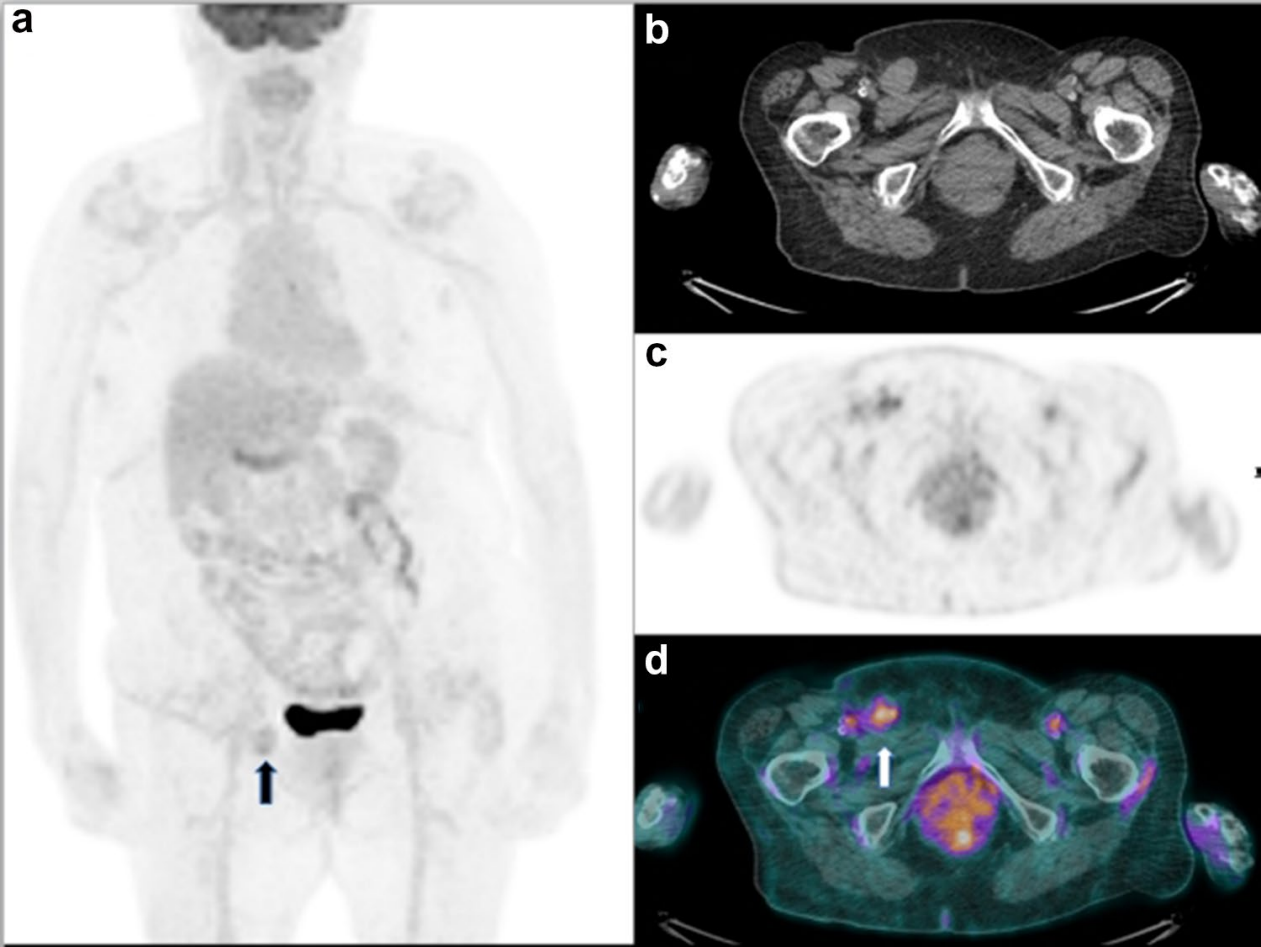
A representative case of false positive ^18^F-FDG PET/CT in 78-year-old woman with a suspected nodal relapse after therapy in a recent pelvic CT. MIP (**a**) showed the presence of a focal uptake in the right inguinal cave. Axial CT (**b**), PET (**c)** and PET/CT fused images (**d**) confirmed the presence of an increased FDG uptake corresponding to a right inguinal node, but after a specific antibiotic therapy the lymph node disappeared

The patient with false positive PET/CT had an increased uptake corresponding to a groin lymph node, but the subsequent biopsy showed the inflammatory nature of this lesion (Fig. 3).

A specific analysis based only on nodal recurrence (*n* 19) showed a SE, SP, PPV, NPV and AC of 100% (CI 81–100%), 98% (CI 88–100%), 95% (CI 72–100%), 100% and 98% (91–100%), respectively. Positive and negative likelihood ratios were 44.00 and 0.00. The average size of nodal recurrences was 17 mm (range 7–32) and the mean SUVmax, SUVmean, L-L SUV R and L-BP SUV R were 12.5, 8.6, 4 and 6.5.

### Clinical impact of PET/CT

A significant impact of ^18^F-FDG PET/CT was detected in 28 patients (44%) showing additional lesions by PET/CT not demonstrated by CI methods (ultrasonography, MRI and/or CT with or without contrast agent according to indication) which had modified the patient management. In the remaining 35 (56%) cases, ^18^F-FDG PET/CT had no clinical impact due to the concordance between PET/CT and other CI examinations. In 12 patients, PET/CT showed the presence of FDG-positive metastatic disease not detected by CI moving from local therapy to chemotherapy; in ten patients in presence of negative CI, PET/CT resulted positive and allowed to start specific therapy; in six patients with suspected disease at CI, 18f-FDG PET/CT confirmed to be true negative (patients remained in “watch and wait” approach and avoided unnecessary invasive treatments).

### Prognostic role of 18F-FDG PET/CT

**Fig. 4:**
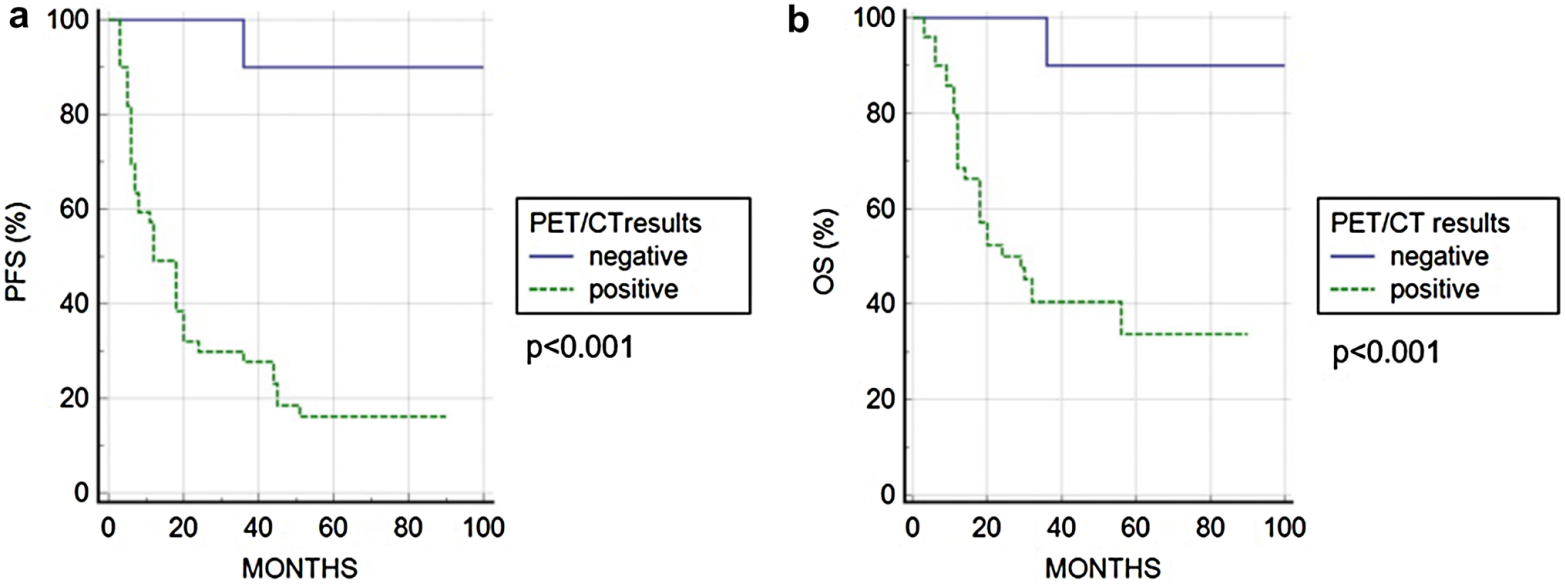
Progression free survival (**a**) and overall survival (**b**) curves according to ^18^F-FDG PET/CT results

After a mean follow-up period of 36 months (range 2–100) from restaging ^18^F-FDG PET/CT, the recurrence or progression of disease happened in 41 (65%) patients with an average time of 15 months (range: 3–51 months), while the death occurred in 30 (48%) patients with an average time of 17.9 months (range 3–56). Considering 51 patients with positive PET/CT, 35 underwent systemic chemotherapy, while 14 undertook local treatment (radiotherapy in 9 cases and surgery in 5). Two patients refused new treatments and were followed with radiological and clinical visits. The median PFS was 18 months, while the median OS 29 months. The estimated 3-year PFS and OS rates were 39% and 49%, respectively. A positive restaging ^18^F-FDG PET/CT scan was significantly correlated with shorter PFS and OS compared to negative restaging PET/CT scan (3-year PFS 26% vs. 91% *p* < 0.001; OS 37% vs. 91% *p* < 0.001) (Fig. 4). Only one patient with negative restaging PET/CT, developed recurrence and death 36 months after PET/CT. In the univariate Cox regression analysis, unremarkable ^18^F-FDG PET/CT scan was associated with a significantly longer survival rate (PFS and OS) compared to a positive scan (Table 2). Similarly, advanced age (> 65 years old) and HPV status (negative) correlated with a shorter PFS and OS. The other main clinical features were not associated with survival. At multivariate analysis, advanced age, negative HPV status and PET/CT positivity were confirmed as the only variables with independent prognostic value, both for PFS and OS (*p* < 0.001 and *p* < 0.001 for age; *p* 0.001 and *p* 0.017 for PET/CT results, respectively).

**Table 2:** Cox regression analysis for the prediction of PFS and O

Variable	PFS			
	Univariate		Multivariate	
	*p* value	HR (95% IC)	*p* value	HR (95% IC)
Age > 65 y	< 0.001	4.213 (2.170–8.180)	< 0.001	6.877 (1.858–16.594)
Stage III–IV(FIGO)	0.623	1.479 (0.312–6.950)		
Histotype squamous cell carcinoma	0.606	1.439 (0.359–5.755)		
HIV infection positive	0.500	1.999 (0.659–7.122)		
HPV infection positive	< 0.001	0.540 (0.229–0.910)	0.003	0.550 (0.255–0.799)
Tumor size	0.209	0.590 (0.261–1.345)		
Lymph-nodal disease at diagnosis	0.684	0.700 (0.151–3.242)		
Interval time between end of therapy and PET/CT	0.356	1.408 (0.570–2.490)		
PET/CT positive	< 0.001	4.137 (2.025–8.453)	0.001	33.592 (3.898–289.487)

	OS			
Variable	Univariate		Multivariate	
	*p* value	HR (95% IC)	*p* value	HR (95% IC)
Age > 65 y	< 0.001	4.244 (1.980–9.095)	< 0.001	5.226 (1.967–13.884)
Stage III–IV(FIGO)	0.601	0.655 (0.134–3.194)		
Histotype squamous cell carcinoma	0.825	1.191 (0.239–9.000)		
HIV infection positive	0.601	1.339 (0.339–5.735)		
HPV infection positive	0.001		0.019	0.434 (0.100–0.820)
Tumor size	0.190	0.533 (0.208–1.367)		
Lymph-nodal disease at diagnosis	0.463	1.808 (0.370–8.8290)		
Interval time between end of therapy and PET/CT	0.501	2.121 (0.759–5.122)		
PET/CT positive	0.003	3.474 (1.493–8.082)	0.017	12.700 (1.555–103.688)

*PFS* progression free survival; *OS* overall survival

## Discussion

Considering the poor prognosis of recurrent VC, the evidence of a helpful way to detect early recurrence is crucial and estimated to have a significant impact on management and survival outcomes. The current recommendation for the evaluation of VC with suspected recurrence involves CI studies such as ultrasonography, CT and/or MRI, but the specificity of these methods is relatively low [15]. ^18^F-FDG PET/CT is a molecular imaging tool that describe the metabolic activity of the tumor, thus could have the potential to overcome the limitations of CI modalities based on morphology.

Our analysis suggests that ^18^F-FDG PET/CT is indeed a valuable modality to workup VC patients with suspected recurrence, probably better than other conventional imaging studies. The diagnostic performances derived by our study was very good with a sensitivity of 100%, a specificity of 92% and an accuracy of 98%. No cases of false negative reports were present and only one false positive uptake at PET/CT then demonstrated as inflammatory nature was reported. Other studies reported similar results in low samples of patients [16–18].

In our study, we analyzed a large population (63 patients) underlining the possible positive impact of PET/CT in detecting local or distant relapse and helping in the management of the patients.

Vargas et al. [19] demonstrated that the combination of MRI and PET could be useful in the study of recurrent mixed gynecological cancers (among them 7 VC), with diagnostic performances better than MR alone or PET/CT.

Moreover, another possible significant impact of ^18^F-FDG PET/CT demonstrated in this study was in the patient management choice. In our analysis, we have demonstrated a positive clinical impact in about 44% of cases. A correct restaging may allow to optimize the management and guide the choice to the more appropriate therapeutic approach, avoiding useless procedures in a significant percentage of patients or guiding to a more effective therapy. In our analysis, we found that in 28 patients, ^18^F-FDG PET/CT results changed the management. In 12 patients, PET/CT switched from local therapy to systemic therapy; in ten patients helped to start specific treatment and in 6 cases continued a “watch and wait” plan avoiding unnecessary invasive approaches. A strength of ^18^F-FDG PET-CT imaging is the whole-body acquisition which allow to study in a single examination the whole body and the hybrid nature of this examination that include both anatomical (CT) and metabolic (PET) data.

Furthermore, PET/CT demonstrated a significant impact also in the prognostic field. Patients with positive restaging PET/CT had significantly shorter PFS and OS compared to patients with negative scans. The relationship between FDG uptake and clinical outcomes has been reported in multiple oncological settings, including primary gynecological cancers [13, 14]. Beside visual analysis also semiquantitative parameters seem to have a potential role in this setting. Our results underline the idea to include PET/CT in restaging field with the additional aim to predict the treatment response and prognosis of VC.

The role of radiomics in the prediction of VC prognosis, although initial [20], seems promising, despite the still standing several methodological issues and open questions. Further studies are needed to confirm on rebut these preliminary evidences.

Based on the findings of this paper, ^18^F-FDG PET/CT seems to be an accurate method for the study and localization of local and distant recurrences in patients affected by VC and demonstrated optimal performances (good sensitivity and specificity) and a positive impact on the clinical decision-making.

An original point of our paper is the analysis of the relationship between HPV status and PET/CT findings. A recent meta-analysis [4] demonstrated that HPV positive VC have a better outcome survival in comparison with HPV negative VC, highlighting a possible different natural history according to the HPV status. Also in our paper, HPV positive women had a better PFS and OS.

However, our study is affected by some limitations: first, the retrospective nature of the study design; second, the absence of histological confirmation of all PET/CT findings (the most reliable method but not ethically or clinically feasible in all cases); third, the relatively low sample of patients analyzed, also due to the relatively uncommon utilization of PET/CT in this relatively rare disease. Despite these encouraging preliminary literature results and our own experience, the definitive role of 18F-FDG PET/CT in recurrent VC should be explored in well-designed multicentre prospective studies with histopathological confirmations to avoid possible bias to controvert or confirm our evidences.

In conclusion, ^18^F-FDG PET/CT demonstrated to be an accurate tool for the study of VC patients with suspected recurrence of disease with a good diagnostic performance and a significant impact on clinical decision-making. Moreover, restaging PET/CT findings are related with prognosis.
